# The Perinatal Multisite Psychiatry Databank: A Cohort Update

**DOI:** 10.3390/ijerph22050684

**Published:** 2025-04-25

**Authors:** Mariane Aumais, Francois Freddy Ateba, Rahel Wolde-Giorghis, Kathelijne Keeren, Barbara Hayton, Sawsan Kalache, Isabelle Collin, Hannah Schwartz, Kirsten Gust, Marie-Josée Poulin, Andréanne Wassef, Katherine Tardif, Martin St-André, Irena Stikarovska, Phyllis Zelkowitz, Catherine M. Herba, Eszter Szekely

**Affiliations:** 1 School of Medicine, Faculty of Medicine and Health Sciences, McGill University, Montréal, QC H3G 2M1, Canada; mariane.aumais.med@ssss.gouv.qc.ca; 2 Lady Davis Institute for Medical Research, Jewish General Hospital, Montréal, QC H3T 1E2, Canada; francois.freddy-ateba@ladydavis.ca (F.F.A.); kathelijne.keeren@ladydavis.ca (K.K.); phyllis.zelkowitz@mcgill.ca (P.Z.); 3 Department of Psychiatry, McGill University, Montréal, QC H3A 1A1, Canada; rahel.wolde-giorghis.med@ssss.gouv.qc.ca (R.W.-G.); barbara.hayton@mcgill.ca (B.H.); sawsan.kalache@mcgill.ca (S.K.); hannah.schwartz@mcgill.ca (H.S.); kirsten.gust@mcgill.ca (K.G.); 4 McGill University Health Center, Glen Site, Montréal, QC H4A 3J1, Canada; 5 Department of Psychiatry and Addictology, Université de Montréal, Montréal, QC H3T 1J4, Canada; andreanne.wassef.med@ssss.gouv.qc.ca (A.W.); martin.st-andre.hsj@ssss.gouv.qc.ca (M.S.-A.); irena.stikarovska.med@ssss.gouv.qc.ca (I.S.); herba.catherine@uqam.ca (C.M.H.); 6 Jewish General Hospital, Montréal, QC H3T 1E2, Canada; isabelle.collin.ccomtl@ssss.gouv.qc.ca; 7 St. Mary’s Hospital, Montréal, QC H3T 0A2, Canada; 8 Institut Universitaire en Santé Mentale de Québec, Centre Intégré Universitaire de Santé et de Services Sociaux de la Capitale-Nationale (CIUSSSCN), Québec, QC G1J 2G3, Canada; marie-josee.poulin.med@ssss.gouv.qc.ca; 9 Centre Hospitalier de l’Université de Montréal, Montréal, QC H2X 0C1, Canada; 10 Douglas Mental Health University Institute, Montréal, QC H4H 1R3, Canada; katherine.tardif.comtl@ssss.gouv.qc.ca; 11 Centre Hospitalier Universitaire Saint-Justine, Montréal, QC H3T 1C5, Canada; 12 Department of Psychology, Université du Québec à Montréal, Montréal, QC H3C 3P8, Canada; 13 Azrieli Research Center of CHU Sainte-Justine, Montréal, QC H1T 1C9, Canada

**Keywords:** peripartum period, pregnancy, postpartum, mental health, anxiety, depression, maternal health services, psychiatry, cohort

## Abstract

The Perinatal Multisite Databank (PMD) aims at facilitating research on perinatal mental health by collecting clinical information of patients referred for evaluations at perinatal mental health clinics across the province of Quebec, Canada with the potential to improve patient care and support evidence-based practice. This study provides a detailed description of the first 693 participants concerning psychosocial risk characteristics, the prevalence of psychiatric disorders and comorbidity during the perinatal period, the evolution of perinatal depression and anxiety symptoms over time, and the treatments received. Data were collected using clinical reports and well-validated questionnaires at multiple timepoints (from pregnancy to 6 months postpartum). Results are discussed within the context of improving patient care and disease prevention strategies in the perinatal period.

## 1. Introduction

The transition to motherhood can be challenging for many women; more than 20% of women in the peripartum period (i.e., during pregnancy and the first postpartum year) experience symptoms of anxiety or depression [1,2,3]. While anxiety and depression are frequent, women with bipolar disorder are also more likely to experience a relapse in the perinatal period [4,5,6,7]. In addition, other psychiatric diagnoses are also more prevalent in this period, such as obsessive–compulsive disorder [8]. Postpartum psychosis, while rare, remains a severe puerperal psychiatric disorder, potentially endangering the life of the woman and her child [9]. Importantly, suicide constitutes one of the leading causes of maternal death in high-income countries [10]. Perinatal mental health problems should be taken seriously since, in addition to affecting women’s well-being and functioning during the important transition to parenthood, they can also influence children’s emotional, cognitive, and social development at various stages of development [11,12,13].

Despite the increased prevalence of psychiatric disorders in the perinatal period and their potentially negative consequences for the whole family, there is a paucity of comprehensive clinical databanks focusing on women’s mental health in the perinatal period. Some of the reasons include challenges in recruiting and retaining pregnant participants due to time and cost constraints, as well as the burden on patients and clinicians, and the large heterogeneity in terms of treatments and treatment outcomes [14]. These difficulties leave many critical research questions unanswered despite the wealth of data collected by physicians during regular clinical appointments. Integrating data collection into clinical settings, with the help of clinicians, provides an efficient and practical solution to these challenges. Utilizing databanks for psychiatric research is relatively inexpensive and has the potential to reduce expenses while enhancing outcomes for both patients and health care providers [15,16]. Leveraging multisite clinical databanks offers significant benefits. It enables the analysis of health records on a larger scale with more diverse samples, enhancing statistical power, generalizability, and the ability to study subtype within heterogeneous diagnostic groups [17]. Furthermore, these databanks facilitate increased collaboration between research sites during longitudinal studies, leading to results that are more comparable through the use of standardized methods and reduced site-specific variations.

Thus, in 2018, the Perinatal Multisite Databank (PMD) was established in Quebec with the aim to facilitate research on perinatal mental health by collecting data on women who receive psychiatric services during the peripartum period. The purpose and development of the PMD has been previously described in detail in Kattan et al. [18]. Briefly, the PMD aims to address four main areas of unanswered research questions concerning perinatal mental health: (1) the factors that differentiate between those with perinatal distress and those with more severe perinatal psychiatric disorders, (2) the course of different perinatal mental disorders, (3) the best treatments and practices across the spectrums of clinical severity and perinatal stage (pregnancy trimesters and postpartum period) for different disorders, and (4) the effects that sociodemographic variables have on treatment adherence and effectiveness [18]. Kattan et al. [18] also presented a demographic overview of the first 225 participants. To date, the number of participants in the databank has more than tripled, totaling 693 participants, and new sites have been added. The goal of the present study is to provide a cohort update of the PMD, one of the few large-scale databanks of perinatal psychiatric patients. Here, we present a detailed description of their sociodemographic and psychosocial risk characteristics, psychiatric history, current rates of diagnoses, types of treatment, and the evolution of their anxiety and depressive symptoms over the perinatal period.

## 2. Materials and Methods

In 2014, a perinatal reproductive psychiatry network was established by researchers and clinicians from six hospital sites that offer perinatal psychiatric services to obstetrical patients in Montreal and Quebec City. These sites included the Jewish General Hospital, Saint-Mary’s Hospital, McGill University Health Center, Hôpital du Sacré-Coeur-de-Montréal, Centre hospitalier universitaire Sainte-Justine, and the Centre intégré universitaire de santé et de services sociaux de la Capitale-Nationale. Some of these sites are specialized in following high-risk obstetrical patients, that is, patients whose pregnancies are more at risk of developing complications. Other sites are community hospitals following patients who have a low risk of developing obstetrical-related complications. Data collection for the PMD began in 2018. In 2023 and 2024, two new sites were added: the Centre hospitalier de l’Université de Montréal and the Douglas Mental Health University Institute. The network’s overarching goal was to create a mechanism to collect standardized patient data for clinical and research purposes, such that information collected during clinical appointments could be stored in a secure databank that protects patient confidentiality. Every attempt was made to find the right balance between burden and questionnaire length for the patient and inclusion of the most relevant information. Permission and scoring manuals were requested from authors when needed, and tools not available in French were translated and back-translated, as mental health services are offered in both French and English in Quebec [18]. The secure electronic databank selected for data collection purposes (REDCap 14.6.9) can collect data offline, function across the different sites, easily export data for analysis, and is hosted by Information Management Service at the Lady Davis Institute for Medical Research affiliated with the Jewish General Hospital in Montréal, Québec, Canada.

This cohort update describes a sample of 693 women who were either pregnant or within six months postpartum at the time of their entry in the study and have met with a perinatal mental health professional at a participating site between July 2018 (the start of the study) and July 2024. A total of 1044 women were invited to participate in the study, indicating a recruitment success rate of 66%. The majority of participants were recruited from the Montreal sites (74%), while over a quarter of the participants have been recruited at our Quebec City site (26%). Inclusion criteria included being pregnant or within 6 months after giving birth to a live baby. Exclusion criteria consisted of being younger than 18 years of age and not being able to communicate in French or English.

Eligible participants were approached before their first consultation with the perinatal mental health professional. Upon their recruitment and consent into the study, participants were asked to complete a set of questionnaires via REDCap 14.6.9 before their first meeting with the perinatal mental health specialist. Questionnaires assessed detailed sociodemographic and psychosocial risk information and anxiety and depressive symptoms. Following the first appointment with the perinatal mental health specialist, the specialists completed a clinician history-taking form that included detailed information about the patient’s personal and familial psychiatric history, current diagnoses, and treatment plan.

Women who enrolled during pregnancy received a set of follow-up questionnaires at 6 weeks postpartum and at 4–6 months postpartum. Women who enrolled in the study postpartum could do so up to six months after giving birth. As such, after their intake questionnaires, they were sent only one set of follow-up questionnaires at 4–6 months postpartum. Since the women recruited postpartum all had given birth at varying times prior to recruitment (theoretically ranging from 1 day to 6 months postpartum), it was not appropriate to send them their follow-up questionnaires at a set time. The timing of follow-up questionnaires for postnatally recruited participants included some flexibility to take into account the time that had elapsed since delivery and their recruitment into the study, while also ensuring enough time between the two postnatal questionnaires (e.g., at least 1 month for those recruited later in the postnatal period).

The timeline and measures to complete by prenatally recruited participants are shown in Table 1 and for postnatally recruited participants in Table 2. Information on the reliability and validity of each measure can be found in Kattan et al. [18].

Continuous variables are described in terms of their mean (M) and standard deviation (SD). Categorical variables are described in terms of their frequencies and percentages (%).

The databank mechanism adheres to the ethical standards set forth in the 1964 Declaration of Helsinki and its subsequent amendments. It was reviewed by the Institutional Review Board of the primary investigating site using their Multicenter Mechanism (reference code: 17-115/MP-05-2018-774), which streamlines the approval process for collaborating sites. Patients provide written informed consent before their data are included in the databank. Participants are assured that the system removes all identifying information, except for email addresses, which are stored in the databank. Email addresses are heavily protected behind several layers of security provided by the software system and can be accessed only by specified members of the research team to contact participants for future phases of the study. Further details can be found in Kattan et al. [18].

## 3. Results

### 3.1. Sociodemographic Characteristics

Of the 693 participants, 522 (75.3%) were pregnant at the time of their entry into the study (Table 1). The age of the participants varied between 19 and 48 years old (M = 32.7 years, SD = 4.8 years). The majority (87.8%) of the participants were married or living with their partner. More than 2/3 of our sample was born in Canada and 1.5% were refugees. Caucasian participants comprised the majority (68.1%) of the sample. Those who identified as being Black, Middle Eastern, Asian, or Hispanic were less than 10% each. Indigenous participants comprised only 1% of the sample. Over 60% of women had a university degree, while 16.3% had a high school diploma or less. Close to half of the participants declared an annual household income of CAD 100,000 or greater. More than half of the women (56.7%) were not currently working, while the remaining 43.3% were employed and working (full- or part-time) at their entry into the study. Detailed sociodemographic characteristics of the databank are shown in Table 3.

### 3.2. Psychiatric History and Current Psychiatric Diagnoses

#### 3.2.1. Personal Psychiatric History

**Figure 1:**
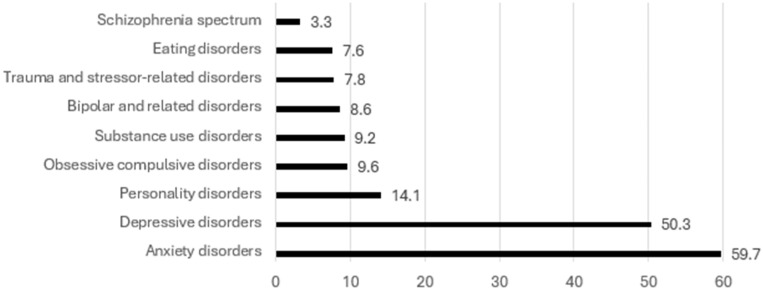
Personal psychiatric history (N = 693).

Anxiety disorder was the most common in patients’ personal psychiatric history (59.7%) closely followed by depressive disorders (50.3%). The least common psychiatric history was schizophrenia spectrum and other psychotic disorders (3.3%), while substance use disorders, bipolar and related disorders, trauma and stressor-related disorders, and eating disorders were all below 10%. Patients may have more than one psychiatric diagnosis. Detailed personal psychiatric history is shown in Figure 1.

#### 3.2.2. Family History of Psychiatric Disorders

**Figure 2:**
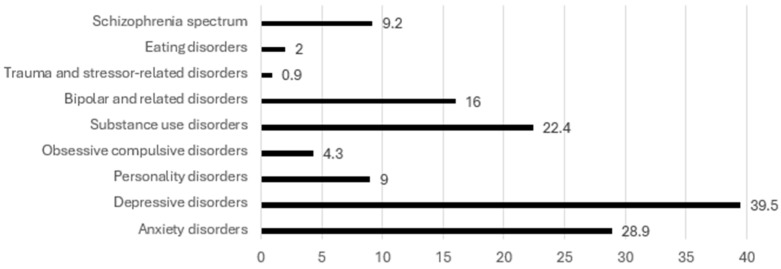
Family history of psychiatric disorder(s) (N = 693).

Similarly to personal psychiatric history, the two most common psychiatric diagnoses in the family history were depressive disorders (39.5%) and anxiety disorders (28.9%). Interestingly, substance use disorders were more common in patients’ family history than personal history (22.4% vs. 9.2%). Many participants also had a family history of bipolar and related disorders (16%) and schizophrenia spectrum disorders (9.2%). Obsessive–compulsive disorders, eating disorders, and trauma and stressor-related disorders were the least common in patients’ family history, all representing under 5% of the sample. Participant’s family may have more than one psychiatric diagnosis. Detailed family history of psychiatric disorder(s) of patients is shown in Figure 2.

#### 3.2.3. Current Psychiatric Diagnoses

Most participants were diagnosed with an anxiety disorder (53.4%; Table 4). The most prevalent anxiety disorder was generalized anxiety disorder with more than a third of the sample having this diagnosis. Depressive disorders were also common (35.6%), particularly major depressive disorder (30.3%). Adjustment disorder was diagnosed in 14.0% of the sample, which is in line with the fact that the population studied is a clinical one. Trauma and stressor-related disorders were diagnosed in 12.0% of the sample. However, only a little over half of those were diagnosed with a posttraumatic stress disorder. Close to 12% of the sample was diagnosed with a personality disorder, oftentimes borderline personality disorder (7.5%) or obsessive–compulsive personality disorder (4.3%). Around 10% of the sample had a diagnosis of obsessive–compulsive and related disorder, and another 10.2% were diagnosed with an attention deficit/hyperactivity disorder. Bipolar disorders were diagnosed in 6.9% of the sample. Less prevalent diagnoses included substance use disorders (5.2%), eating disorders (3.9%), schizophrenia spectrum and other psychotic disorders (2.5%), and somatic disorders (1.2%).

**Figure 3:**
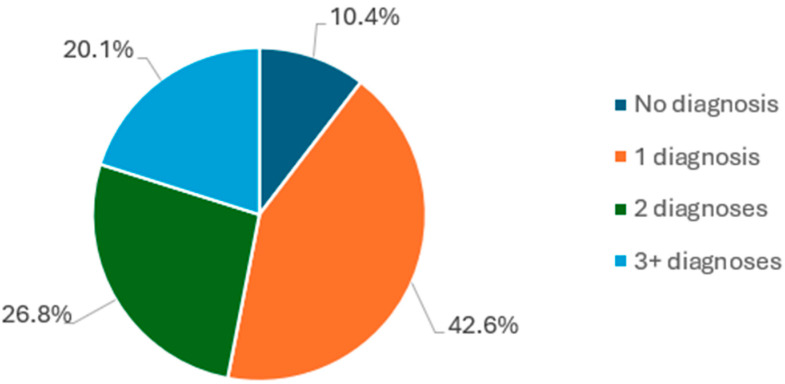
Number of comorbidities.

Comorbidity was common in the sample. At intake, close to half of the sample had more than one diagnosis (46.9%), and one in five participants (20.1%) had three or more diagnoses (Figure 3).

### 3.3. Treatments

Both non-pharmacological and pharmacological treatments were suggested by the perinatal mental health professionals, however, non-pharmacological treatments were prioritized over pharmacological treatments, with 70.7% of the sample being recommended psychotherapy (Table 5). Moderate physical activity was recommended to 12.4% of the sample and medical leave was prescribed to 8.9%. Among the different pharmacotherapies, antidepressants were most often prescribed (38.5%). Other medications prescribed included antipsychotics (11.1%), benzodiazepines (6.1%), non-benzodiazepine hypnotics (1.0%), mood stabilizers (0.9%), and stimulants (0.6%).

### 3.4. The Course of Perinatal Depressive and Anxiety Symptoms

#### 3.4.1. Depressive Symptoms

**Figure 4:**
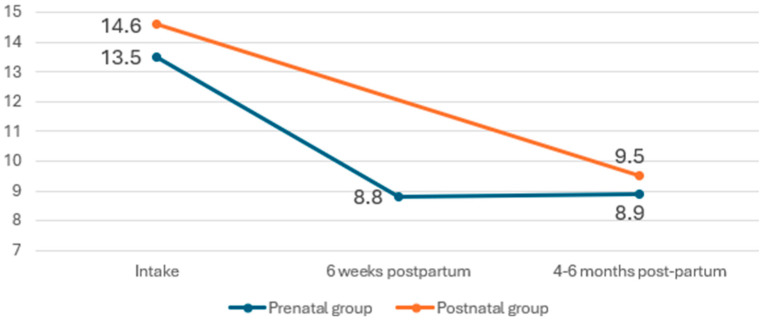
Depressive symptoms (EPDS scores) at the different assessment points.

At study entry, both the prenatally and postnatally enrolled participants had high levels of depressive symptoms, with a mean EPDS score above 13 in both groups, indicating a high likelihood of clinical depression (Figure 3). The prenatal group was assessed at intake (i.e., anytime during pregnancy), 6 weeks postpartum, and 4–6 months postpartum. The postnatal group was assessed at intake (i.e., 0–6 months postpartum) and at 4–6 months postpartum (where applicable). The cutoff score for clinically relevant depression varies between studies but 13 is considered a widely used threshold [19]. With time, both groups showed a decreasing trend with EPDS scores around 9 at the 4–6-month follow-up (Figure 4).

#### 3.4.2. Anxiety Symptoms

**Figure 5:**
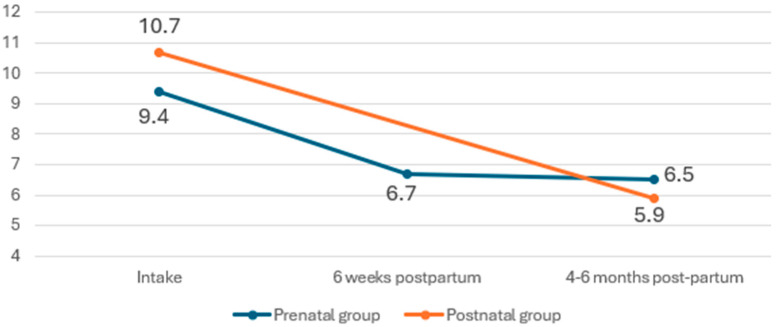
Anxiety symptoms (GAD-7 scores) at the different assessment points.

The prenatal group was assessed at intake (i.e., anytime during pregnancy), 6 weeks postpartum, and 4–6 months postpartum. The postnatal group was assessed at intake (i.e., 0–6 months postpartum) and at 4–6 months postpartum (where applicable). Prenatally recruited participants had on average mild levels of anxiety at intake (GAD-7 M = 9.4, SD 5.4), while postnatally recruited participants had moderate levels of anxiety (M = 10.7, SD = 5.6), given the commonly used GAD-7 cutoff bands of 5–9 and 10–14 indicating mild and moderate anxiety, respectively [20] (Figure 5). Similarly to depressive symptoms, anxiety symptoms also improved during the postnatal period and remained mild in both prenatally and postnatally recruited participants (Figure 5).

## 4. Discussion

This cohort update provides a detailed description of the clinical, sociodemographic, and psychosocial characteristics of participants in the Perinatal Multisite Databank, a unique clinical cohort of patients who received perinatal psychiatric services in Quebec.

Most of the demographic and psychosocial characteristics are in line with those generally observed in the province of Quebec, indicating that a clinical population of women in the perinatal period may not vary significantly, at least on the studied characteristics, from the general population of women in the same age range. However, some results are noteworthy, including the proportion of women born in Canada. According to Statistics Canada, in 2021, 76% of women aged 20–49 years living in Quebec were born in Canada [21], while only 68% of our participants were Canadian born. This may suggest that our sample is somewhat more diverse than the general population in the province of Quebec. However, in the city of Montreal, which is where most of our recruitment took place (six of our seven sites), there are twice as many immigrants compared to the rest of the province [22]. In light of this, it is not surprising that immigrant women are slightly over-represented in our sample.

Another noteworthy result is the high prevalence of highly educated women in our sample, especially given that recruitment was performed in a clinical context. A lower educational level is considered a risk factor for developing perinatal mental health disorders [23,24]. Nevertheless, women living in Montreal are generally more highly educated with approximately 55.5% having a university degree in 2023 [25]. Thus, our results may be partly explained by the fact that larger proportions of Montreal residents tend to study in higher education than residents in the rest of the province of Quebec. It is likely that the educational composition of our sample will change, as more participants residing outside of Montreal will participate in the databank.

Anxiety and depressive disorders are the most prevalent diagnoses among mental health problems in the perinatal period (~20%) [7], which was also reflected in our databank. An important risk factor for perinatal depression is having a personal history of depression, potentially increasing the risk of developing postpartum depression by 20 times [26]. Moreover, a family history of psychiatric disorders and the presence of a comorbid psychiatric disorder are further important risk factors for developing an anxiety disorder in the perinatal period [23].

We observed that 46.9% of databank participants had more than one concomitant diagnosis, and, among participants who had a diagnosis (not everyone met criteria for a clinical diagnosis), more than half (52%) presented comorbidities. This overlaps with the lower end of the range that is generally reported for psychiatric comorbidities outside the perinatal period (12-month comorbidity between 50 and 70%) [27,28]. Data regarding the prevalence of psychiatric comorbidities in the perinatal period are scarce. One recent study found that 43% of their participants with a perinatal psychiatric diagnosis had more than one diagnosis [29]. This figure is lower than our findings, likely due to the authors’ methodological decision to consider only four DSM diagnoses alongside suicide risk.

As per the American Psychiatric Association guidelines, clinicians must balance the risks of an untreated major depressive disorder in pregnant women and the risks of using antidepressants [30]. Accordingly, non-pharmacological treatments (e.g., psychotherapies) should be part of the first-line treatment for mild to moderate depression during this period if patients are open to such treatment and it is accessible in terms of cost and wait times. Similarly, Canadian guidelines on the treatment of anxiety disorders advocate for the use of psychotherapy or antidepressant medication [31]. While the natural course of perinatal anxiety disorders tends to decrease in severity and prevalence over time [32], the natural course of perinatal depression tends to remain stable over time [33]. Mental health specialists involved in the PMD followed the above guidelines and our findings showed consistent improvements in both anxious and depressive symptoms of patients over the perinatal period.

Despite the important strengths of the databank, this study has limitations. While a mental health specialist interviewed the participants and provided clinical diagnoses, a large proportion of additional information was self-reported, such as psychosocial risk and anxiety and depressive symptoms. Furthermore, most of the participants to date have been recruited in Montreal, potentially biasing the results in favor of a representation of this urban population rather than the population of the entire province, limiting the generalizability of our findings. However, this limitation will be attenuated as the PMD grows and new sites will be included from other areas of the province (e.g., the Centre intégré de santé et de services sociaux des Laurentides), which will make future results more generalizable to the population of Quebec. Additionally, while the perinatal period is often considered to continue up to one year postpartum, our data collection was limited to six months postpartum due to feasibility constraints.

Avenues for future research in perinatal mental health might include considering studying the impact of social determinants of health on women’s mental health and collecting data on perinatal mental health up to one year after giving birth.

## 5. Conclusions

The PMD is the only databank in Quebec collecting data on mental health in a clinical sample during the perinatal period, prospectively and across multiple sites. The present study adds significant information to the current landscape of perinatal psychiatry services by presenting characteristics of a large perinatal psychiatric sample, as well as data on risk factors, prevalence of diagnoses, psychiatric history, comorbidities, treatments, and the evolution of anxious and depressive symptoms during the perinatal period. The PMD also provides rich data for research collaborations in the field of perinatal mental health.

## Figures and Tables

**Table 1:** Timeline of protocol 1 (first seen during pregnancy).

Measure	Intake	6 Weeks Postpartum	4–6 Months Postpartum
Contact information and demographics—full version	✓		
Questions on exercise	✓	✓	✓
Questions on complementary alternative medicine (CAM)	✓		
Newborn and birth information		✓	
Demographics—update		✓	✓
The Edinburgh Postnatal Depression Scale (EPDS)	✓	✓	✓
The Generalized Anxiety Disorder (GAD)-7	✓	✓	✓
The Antenatal Risk Questionnaire (ARNQ)—full version	✓		
Postnatal Risk Questionnaire (PRNQ)		Last 3 questions ✓	Full version ✓
The Cambridge Worry Scale (CWS)	✓		
The Relationship Questionnaire (RQ)	✓		
The Adverse Childhood Experiences Questionnaire (ACE)	✓		
The Postpartum Bonding Questionnaire (PBQ)		✓	✓
Pregnancy Risk Assessment Monitoring System Questionnaire (PRAMS)—14 questions		✓	✓
The Barkin Index of Maternal Functioning (BIMF)		✓	✓
Clinician history-taking form	✓		
Quality assurance questionnaire (added in June 2021)		✓	✓
COVID-19 module (between October 2020 and February 2024)	✓		

✓ A check mark signifies the questionnaire was provided to the participant.

**Table 2:** Timeline of protocol 2 (first seen after delivery).

Measure	Intake	4–6 Months Postpartum
Contact information and demographics—full version	✓	
Questions on exercise	✓	✓
Questions on complementary alternative medicine (CAM)	✓	
Newborn and birth information	✓	
Demographics—update		✓
The Edinburgh Postnatal Depression Scale (EPDS)	✓	✓
The Generalized Anxiety Disorder (GAD)-7	✓	✓
Postnatal Risk Questionnaire (PRNQ)—full	✓	✓
The Relationship Questionnaire (RQ)	✓	
The Adverse Childhood Experiences Questionnaire (ACE)	✓	
The Postpartum Bonding Questionnaire (PBQ)	✓	✓
Pregnancy Risk Assessment Monitoring System Questionnaire (PRAMS)—14 questions	✓	✓
The Barkin Index of Maternal Functioning (BIMF)	✓	✓
Clinician history-taking form	✓	
Quality assurance questionnaire (added in June 2021)		✓
COVID-19 module (between October 2020 and February 2024)	✓	

✓ A check mark signifies the questionnaire was provided to the participant.

**Table 3:** Sociodemographic characteristics of the PMD to date (N = 693).

Variable	Variable Choice	n (%)
Pregnancy status at intake (N = 693)	Pregnant	522 (75.3)
	Postpartum	171 (24.7)
Age (n = 609), years	Mean (SD)	32.7 (4.8)
	Minimum	19
	Maximum	48
Parity (n = 502)	Current one is their first pregnancy/baby	179 (35.7)
	One or more prior deliveries	323 (64.3)
Marital status (n = 682)	Married or living with partner	599 (87.8)
	Single, not living with partner, separated, divorced, or widowed	83 (12.2)
Immigration status	Canadian citizen ^†^	578 (84.9)
(n = 681)	Permanent resident	66 (9.7)
	Temporary resident	27 (4.0)
	Refugee	10 (1.5)
Ethnicity * (N = 693)	Caucasian	472 (68.1)
	Black	56 (8.1)
	Middle Eastern	40 (5.8)
	Asian	46 (6.6)
	Hispanic	34 (4.9)
	Indigenous	7 (1.0)
	Other	58 (8.4)
Education (n = 681)	High school or lower	111 (16.3)
	CÉGEP **	144 (21.1)
	Bachelor’s	254 (37.3)
	Master’s	138 (20.3)
	Doctorate	34 (5.0)
Annual household	Under CAD 39,999	115 (17.1)
income (n = 674)	CAD 40,000–59,999	80 (11.9)
	CAD 60,000–79,000	63 (9.3)
	CAD 80,000–99,999	102 (15.1)
	CAD 100,000–120,000	106 (15.7)
	Over CAD 120,000	208 (30.9)
Work status	Currently working	295 (43.3)
(n = 681) ***	- Employed full-time	250 (85.6)
	- Employed part-time	42 (14.4)
	Not currently working *	386 (56.7)
	- Full-time homemaker	35 (5.1)
	- Student	27 (3.9)
	- Maternal leave	184 (26.6)
	- Medical leave	108 (15.6)
	- Unemployed	48 (6.9)

^†^ Of the participants who are Canadian citizens, 109 (16.0%) were born outside of Canada. * Categories do not add up to 100% because some participants endorsed multiple options. ** Collège d’enseignement general et professionnel (general and professional teaching college). In Quebec, Canada, it is a school that provides the first level of post-secondary education. *** Missing variables ranged from 0% to 1.0%. No inferential analyses were performed to compare subgroups. Due to rounding, subcategories may not add up to 100%.

**Table 4:** Current psychiatric diagnoses (N = 693).

Psychiatric Diagnosis	N (%)
Anxiety disorders	370 (53.4)
- Generalized anxiety disorder	262 (37.8)
- Panic	60 (8.7)
- Social anxiety	53 (7.6)
- Specific phobia	37 (5.3)
- Other anxiety disorders	102 (14.7)
Depressive disorders	247 (35.6)
- Major depressive disorder	210 (30.3)
- Other depressive disorders	62 (8.9)
Adjustment disorder	97 (14.0)
Trauma and stressor-related disorders	83 (12.0)
- Posttraumatic stress disorder	48 (6.9)
- Other trauma and stressor-related disorders	35 (5.1)
Personality disorders	81 (11.7)
- Borderline	52 (7.5)
- Obsessive–compulsive	30 (4.3)
- Other personality disorders	12 (1.7)
Obsessive compulsive and related disorders	72 (10.4)
Attention deficit/hyperactivity disorder	71 (10.2)
Bipolar and related disorders	48 (6.9)
- Bipolar type I	24 (3.5)
- Bipolar type II	16 (2.3)
- Other bipolar and related disorders	8 (1.2)
Substance use disorder	36 (5.2)
Eating disorders	27 (3.9)
- Anorexia nervosa	13 (1.9)
- Other eating disorders	13 (1.9)
Schizophrenia spectrum and other psychotic disorders	17 (2.5)
- Schizophrenia	7 (1.0)
- Schizoaffective	6 (0.9)
- Other schizophrenia spectrum and other psychotic disorders	5 (0.7)
Somatic disorders	8 (1.2)

Categories do not add up to 100% as participants can have multiple psychiatric diagnoses.

**Table 5:** Non-pharmacological and pharmacological treatments prescribed to PMD participants.

Treatment Type	N (%)
Psychotherapy	490 (70.7)
Moderate physical activity	86 (12.4)
Medical leave	62 (8.9)
Antidepressant	267 (38.5)
Antipsychotic	77 (11.1)
Benzodiazepine	42 (6.1)
Mood stabilizer	6 (0.9)
Non-benzodiazepine hypnotics	7 (1.0)
Stimulants	4 (0.6)

Categories are not exclusive.

## Data Availability

The data presented in this study are available on request from the corresponding author due to ethical reasons.

## Abbreviations

The following abbreviations are used in this manuscript:

PMD	Perinatal Multisite Databank
EPDS	Edinburgh Postnatal Depression Scale
GAD	Generalized Anxiety Disorder
